# Photocatalytic Decolorization and Biocidal Applications of Nonmetal Doped TiO_2_: Isotherm, Kinetic Modeling and In Silico Molecular Docking Studies

**DOI:** 10.3390/molecules25194468

**Published:** 2020-09-29

**Authors:** Muhammad Saqib Khan, Jehanzeb Ali Shah, Muhammad Arshad, Sobia Ahsan Halim, Ajmal Khan, Ahson Jabbar Shaikh, Nadia Riaz, Asim Jahangir Khan, Muhammad Arfan, Muhammad Shahid, Arshid Pervez, Ahmed Al-Harrasi, Muhammad Bilal

**Affiliations:** 1 Department of Environmental Sciences, COMSATS University Islamabad, Abbottabad Campus, Abbottabad 22060, Pakistan; muhammadsaqib@yahoo.com (M.S.K.); jehanzeb360@yahoo.com (J.A.S.); nadiariazz@gmail.com (N.R.); asimjk@cuiatd.edu.pk (A.J.K.); pervez@cuiatd.edu.pk (A.P.); 2 Institute of Environmental Sciences and Engineering (IESE), SCEE, National University of Sciences and Technology, Islamabad 44000, Pakistan; marshad@iese.nust.edu.pk; 3 Natural and Medical Sciences Research Center, University of Nizwa, P.O. Box 33, Birkat Al Mauz, Nizwa 616, Oman; sobia_halim@unizwa.edu.om (S.A.H.); ajmalkhan@unizwa.edu.om (A.K.); 4 Department of Chemistry, COMSATS University Islamabad, Abbottabad Campus, Abbottabad 22060, Pakistan; ahson@cuiatd.edu.pk; 5 Department of Chemistry, SNS, National University of Sciences and Technology, Islamabad 44000, Pakistan; marfan@sns.nust.edu.pk; 6 Department of Environmental Sciences, COMSATS University Islamabad, Vehari Campus, Vehari 61100, Pakistan; muhammadshahid@ciitvehari.edu.pk

**Keywords:** titanium dioxide, water disinfection, photo-biocidal activity, reaction kinetics, molecular docking simulation

## Abstract

Textile dyes and microbial contamination of surface water bodies have been recognized as emerging quality concerns around the globe. The simultaneous resolve of such impurities can pave the route for an amicable technological solution. This study reports the photocatalytic performance and the biocidal potential of nitrogen-doped TiO_2_ against reactive black 5 (RB5), a double azo dye and *E. coli*. Molecular docking was performed to identify and quantify the interactions of the TiO_2_ with β-lactamase enzyme and to predict the biocidal mechanism. The sol-gel technique was employed for the synthesis of different mol% nitrogen-doped TiO_2_. The synthesized photocatalysts were characterized using thermal gravimetric analysis (TGA), scanning electron microscopy (SEM), X-ray diffraction (XRD), Fourier transform infrared spectroscopy (FT-IR), Brunauer–Emmett–Teller (BET) and diffuse reflectance spectroscopy (DRS). The effects of different synthesis and reaction parameters were studied. RB5 dye degradation was monitored by tracking shifts in the absorption spectrum and percent chemical oxygen demand (COD) removal. The best nanomaterial depicted 5.57 nm crystallite size, 49.54 m^2^ g^−1^ specific surface area, 11–40 nm particle size with spherical morphologies, and uniform distribution. The RB5 decolorization data fits well with the pseudo-first-order kinetic model, and the maximum monolayer coverage capacity for the Langmuir adsorption model was found to be 40 mg g^−1^ with K_ads_ of 0.113 mg^−1^. The LH model yielded a higher coefficient K_C_ (1.15 mg L^−1^ h^−1^) compared to the adsorption constant K_LH_ (0.3084 L mg^−1^). 90% COD removal was achieved in 60 min of irradiation, confirmed by the disappearance of spectral peaks. The best-optimized photocatalysts showed a noticeable biocidal potential against human pathogenic strain *E. coli* in 150 min. The biocidal mechanism of best-optimized photocatalyst was predicted by molecular docking simulation against *E. coli* β-lactamase enzyme. The docking score (−7.6 kcal mol^−1^) and the binding interaction with the active site residues (Lys315, Thr316, and Glu272) of β-lactamase further confirmed that inhibition of β-lactamase could be a most probable mechanism of biocidal activity.

## 1. Introduction

Environmental challenges are linked with toxic and carcinogenic recalcitrant contaminants, e.g., textile azo dyes. These noxious water impurities provide the impetus to the fundamental, sustained, and applied research in the area of environmental detoxification. The presence of these contaminants in industrial wastewater is a significant perturbance to the ecological health and the ecosystem, because of their toxicity and resistance to natural degradation processes. The textile industry is one of the significant consumers of water, dyes, and other chemicals, which are used during different stages of fabric processing. Subsequently, a substantial amount of effluents loaded with spent or unutilized dyes, are likely to be dumped in the freshwater ecosystem, if not treated properly. Thus, among various contaminants in textile wastewater, colorants are unquestionably regarded as the most peremptory source of contamination [1].

Titanium dioxide (TiO_2_) is one of the most reported materials in environmental remediation such as the degradation of organic pollutants, air purification, and antibacterial applications [2,3,4,5]. Heterogeneous photocatalysis has been explored as a potential solution for wastewater remediation. Since the study of Fujishima and Honda in 1972 on the splitting of water through TiO_2_ electrodes [6], photocatalytic decolorization of environmental pollutants, especially in wastewater, have extensively been investigated. The TiO_2_-based photocatalyst has considerable potential to disinfect or inactivate the harmful pathogens (*E. coli*). Currently, many approaches have been studied to improve its antibacterial activity. Most of the antibacterial activity of TiO_2_ based photocatalysts has been reported under the ultraviolet region of the spectrum [7,8]. Redox reaction between adsorbed species (water and oxygen) and photogenerated e^-^ and h^+^ produces reactive oxygen species (ROS). Generation of ROS primarily contributes to the disinfection property of TiO_2_ by damaging cell proteins, membrane and nucleic acid. Previously, the major cause of the bactericidal effect was thought to be OH radicals [9] and later h^+^ were also reported for direct oxidation [10]. Keeping in view the cell structure, the first barrier is the outer membrane (Gram negative bacteria), once damaged, the cytoplasmic membrane is disrupted, leading to cell death. Despite the widely reported application of N-doped TiO_2_ catalyst with biocidal capabilities, it exhibits poor antibacterial performance in the visible light region [11].

The effective utilization of TiO_2_ is hindered by its large bandgap (3–3.2 eV) and activation requirements in the UV region of the spectrum. Thus it is essential to tune the bandgap of TiO_2_ into the visible area [12] through modification of the TiO_2_ lattice structure [13,14,15]. Generally, heterogeneous photocatalysis follows the advance oxidation process at the surface of a semiconductor through the absorption of photons, which cause electron excitation from the valance band to the conduction band, and this charge separation leads to the generation of free radicals. Hydroxyl radicals (OH•) are more effective in destroying the organic pollutants in wastewater due to their reactive electrophilic property, non-selectivity, and rapid reaction [16]. The 2.33 V oxidation potential makes them prominent among conventional oxidants like H_2_O_2_ or KMnO_4_ [17]. Furthermore, modifications of the TiO_2_ can significantly improve the disinfection potential as well as the overall performance of the material. TiO_2_ band gap is narrowed through doping of transitional metal cations by the creation of oxygen vacancies or replacement of lattice oxygen with anionic dopants like C, N, B or S dopant, however, substitutional doping of nitrogen is considered more effective in decreasing the bandgap through mixing of N and O 2p states [18,19].

The current investigation focuses on the synthesis, characterization, decolorization efficiency (adsorption and photocatalytic decolorization) for RB5 double azo group dye, and estimation of the dye disappearance kinetics of the nitrogen-doped TiO_2_. Moreover, the best material was further tested for photo-biocidal activity against selected bacterial strain for possible application as a water disinfectant. Furthermore, Molecular docking was performed to identify and quantify the interactions of the N-doped TiO_2_ with *E. coli* and to predict the biocidal mechanism. Multiple target proteins, like β-lactamase, were chosen as a potential focus with active site residues.

## 2. Results and Discussion

### 2.1. Photocatalyst Characterization

#### 2.1.1. Thermal Stability and Suitable Temperature Selection

**Figure 1:**
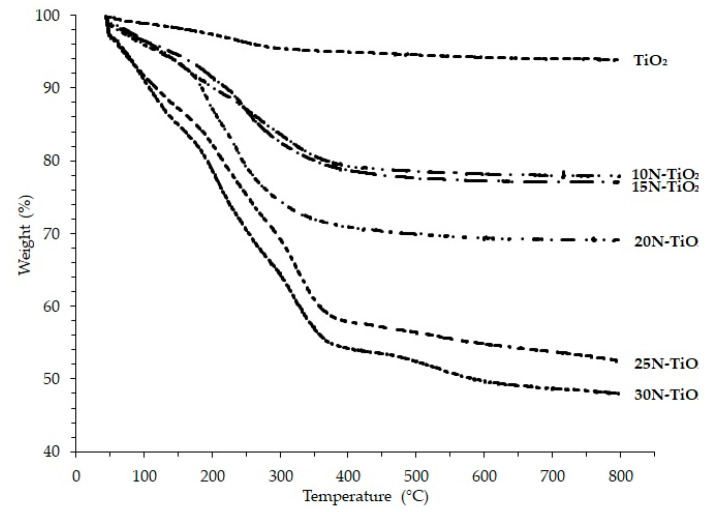
Thermograms of TiO_2_ and N–TiO_2_ photocatalysts.

The appropriate calcination temperature was selected through TGA analysis. Thermograms of various mol % NTiO_2_ shown in Figure 1 displayed the two weight loss steps. This can be attributed to the loss of water residue on the material surface, the burning of organic residue and anatase crystallization in the later phase, the same results were reported in previous work [20].

The weight loss of the raw photocatalysts at different temperature ranges can be shown in Table 1, less than 20% weight decrease as temperature increased from ambient to 150 °C. This is attributed mainly to the loss of physically adsorbed water on the surface of the photocatalyst. This loss in weight of material increased more with an increase in N content and temperature beyond 300 °C; however, weight loss gets slower above 400 °C. Therefore, the calcination temperatures were chosen up until 400 °C, i.e., 200, 300, 400 °C because the loss of nitrogen occurs if the temperature is raised above 400 °C. This finding is supported elsewhere [21].

#### 2.1.2. Surface Morphology

**Figure 2:**
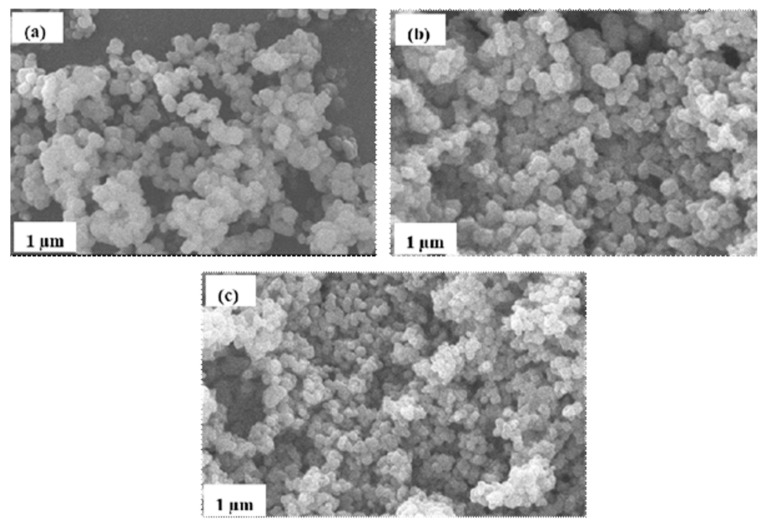
Scanning electron microscopy (SEM) micrographs of (**a**) bare-TiO_2_, (**b**) 20N–TiO_2_-raw and (**c**) 20N–TiO_2_ calcined at 300 °C.

The catalytic properties of a semiconductor can best be explained with the photocatalyst’s morphology, as it plays an important role in its decolorization performance. Figure 2 shows micrographs of bare TiO_2_, 20N–TiO_2_ raw, and 20N–TiO_2_ calcined at 300 °C. The micrographs depict spherical morphologies with uniform distribution. The photocatalysts samples composed of clusters containing nanoparticle composite adhere to each other. The mean size found was around 11–40 nm.

#### 2.1.3. Phase Identification and Crystal Size

**Figure 3:**
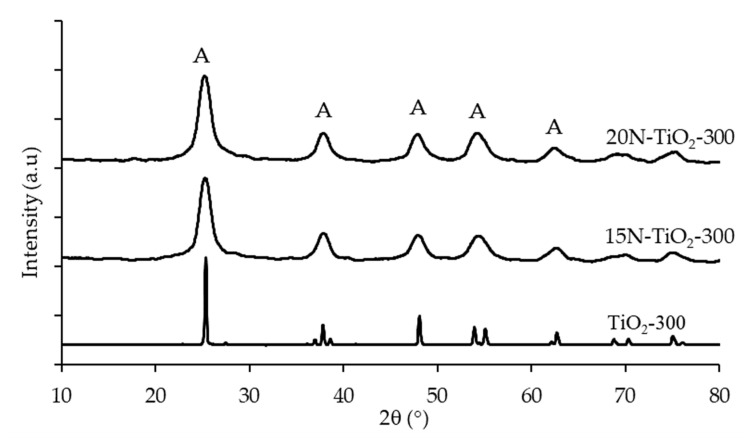
X-ray diffraction (XRD) pattern of anatase TiO_2_ and N–TiO_2_ photocatalysts calcined at 300 °C (A = Anatase phase).

Figure 3 shows the X-ray diffraction peaks of pure anatase TiO_2_, 15N–TiO_2_-300 and 20N–TiO_2_-300 photocatalysts. The diffraction peaks are well assigned to the anatase TiO_2_ crystalline phase (JCPDS 84-1286). Moreover, no peaks for the rutile and brookite phases were detected in 15N–TiO_2_-300 and 20N–TiO_2_-300. The absence of the rutile phase can be ascribed to the lower calcination temperature because many studies reported the transformation of anatase to rutile phase at a calcination temperature of above 600 °C [5,22]. While a calcination temperature of 300 °C was used in this study, the synthesized material has a significant amount of amorphous phase, which represents a major drawback in terms of photocatalytic activity. For comparative purposes, the XRD pattern of the synthesized TiO_2_ is included in Figure 3.

XRD peak intensities of the anatase phase of TiO_2_ become weaker and broader upon doping with nitrogen, which indicates the formation of TiO_2_ crystals. It is also observed that doped nitrogen onto TiO_2_ maintained the pure anatase phase and did not influence the sol-gel synthesized TiO_2_ crystal phase. The same results were obtained for the low-temperature impregnation process of Fe–N/TiO_2_ [23]. The anatase crystallite sizes of each synthesized photocatalysts were calculated through the Scherrer equation (Table 2). The FWHM of anatase (2θ = 25.4°) represents the (1 0 1) plane diffraction, and the broad XRD peaks suggest the occurrence of small crystallite with the average size of 6.28 nm and this is also reported by [24]. The findings of Xing and co-workers are in agreement with our results that doping nitrogen with TiO_2_ reduces the particle size of the photocatalysts [25]. These results also indicate doping N retrains the growth of TiO_2_ crystallite and forestall the transformation of anatase to rutile crystalline phase. The probable reasons could be, either the concentration of N doping is lower than the detection limit by XRD or nitrogen is difficult to be detected in the TiO_2_ framework.

#### 2.1.4. Functional Group Analysis

**Figure 4:**
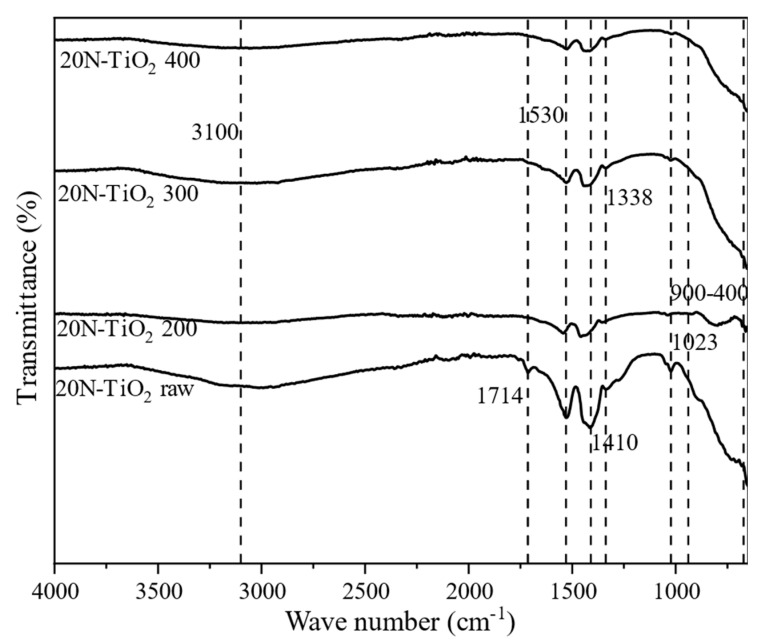
Fourier-transform infrared spectroscopy (FTIR) spectra of as-synthesized and calcined 20N–TiO_2_ photocatalysts.

Figure 4 shows the FTIR spectra of synthesized raw and calcined 20N–TiO_2_-300 in the wavenumber range of 4000–400 cm^−1^. The IR band observed from 400–900 cm^−1^ corresponds to Ti–O stretching vibrations [26,27]. Two dominant transmittance regions at 3000–3200 cm^−1^ and 1000–1700 cm^−1^ were observed in a raw photocatalyst. The broad peak located at 3100 cm^−1^ is due to the stretching of the –OH group, while a sharp peak at 1714 cm^−1^ is associated with –OH mode of water on the surface of TiO_2_ [28]. Interestingly peaks at 3100 cm^−1^ and 1714 cm^−1^ disappeared from raw photocatalysts as the calcination temperature increased; this can be justified in terms of –OH loss from the surface of TiO_2_. The –C–H stretching vibration was observed at 1410 cm^−1,^ which corresponds to residual organic species from precursor alkoxide [29]. Figure 4 also revealed the apparent peaks at 1023 cm^−1^ and 1338 cm^−1^, respectively, due to the presence of N_2_O_2_^−2^ and NO^-^ species [4,29]. The intensity of these two peaks decreased as the calcination temperature increased, which shows the decomposition of N_2_O_2_^−2^ and NO^−^ species at higher calcination temperatures.

#### 2.1.5. BET Surface Area Analysis

**Figure 5:**
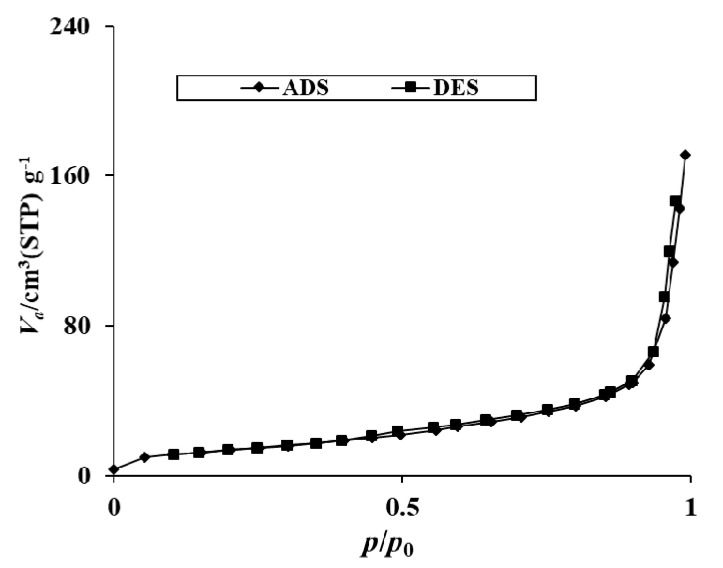
N_2_ adsorption/desorption isotherms of the 20N–TiO_2_-300 photocatalyst.

The textural parameters from the nitrogen adsorption and desorption experiment of 20N–TiO_2_-300 were collected and presented in Table 3 and Figure 5. It is evident from Table 3 that with an increase in nitrogen content onto TiO_2_, surface area decreased from 66.31 to 49.54 m^2^ g^−1^, and pore diameter increased from 6.58 to 21.34 nm. The pore diameter of 20N–TiO_2_-300 clearly shows the mesoporous structures, while bare TiO_2_ shows microporous structures with a high surface area. The pore diameter increases by a factor of 3.1 compared to bare TiO_2_. Results from photocatalysis studies show percent decolorization increases with increase in nitrogen content onto TiO_2_ which suggest that surface area may not be the major contributor in photocatalytic activity but increase in photocatalytic performance can be associated to easy reach of large RB5 dye molecules to the surface of N–TiO_2_ due to larger pore diameter. Nitrogen adsorption–desorption gave a BET type II with H3 hysteresis loop indicating 20N–TiO_2_-300 as a mesoporic materials (average pore diameter = 21.3 nm) and small hysteresis at higher relative pressures indicates capillary condensation. Such mesopores of 20N–TiO_2_-300 include a combination of monolayer-multilayer adsorption and capillary condensation. Previous studies have shown the favored photocatalytic decolorization because of high adsorption capacity which has been associated to the porosity of photocatalysts [30,31].

#### 2.1.6. DRS Bandgap Analysis

**Figure 6:**
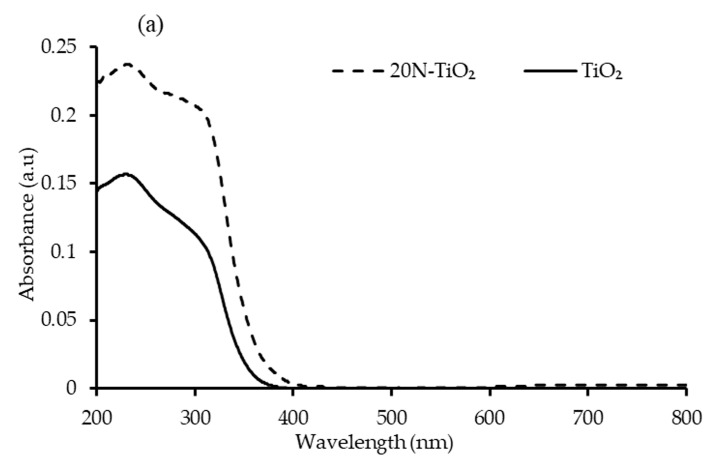

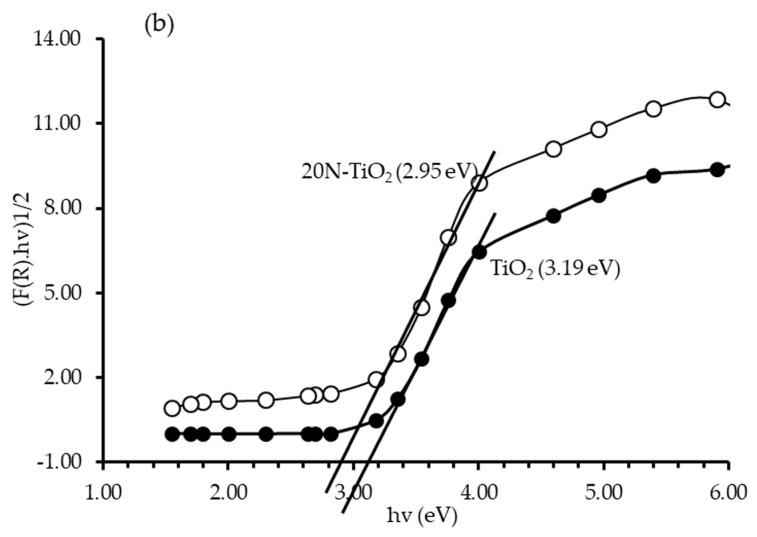
(**a**) Absorbance spectrum of TiO_2_ and 20N–TiO_2_ -300 (**b**) Tauc’s plot for bandgap estimation.

The challenge of enabling TiO_2_ to function in the visible light region is to shift its absorption spectrum from the UV region by adding nonmetallic moieties, and this shift can be clearly seen in Figure 6a. A dominant red shift in the peak of N–TiO_2_ was observed compared to bare TiO_2_ with slight visible-light absorption [32]. A sharp absorption edge of about 390 nm attributed to the electron’s excitation from the VB to CB [33]. Bandgap energy of the photocatalysts determined from the plot of (F(R).hν)^1/2^ against hν. Extrapolating it to the tangent of the graph in the low energy range (hv) axis yields the semiconductor bandgap when [F(R).hv]^1/2^ = 0 [34]. The bandgap obtained in this way was 3.19 eV for TiO_2_ and 2.95 eV for 20N–TiO_2_-300, indicating the noticeable structural modification of TiO_2_ with nitrogen, thus enhancing the photon absorption properties [35], and the bandgap estimation can be seen in Figure S2.

### 2.2. Photodecolorization Studies

#### 2.2.1. Screening Studies for Calcination Temperature and Nitrogen Loading

Table 4 depicts the effect of different calcination temperatures of 200 °C, 300 °C and 400 °C and mol% nitrogen loading of 0, 5, 10, 15, 20, 25 and 30, on the decolorization of RB5 at a solution pH of 6.8. During one hour of the reaction, 0N–TiO_2_ showed 41%, 45% and 30% decolorization for RB5 dye at calcination temperatures of 200 °C, 300 °C and 400 °C, respectively. Similarly, 15N–TiO_2_ and 30N–TiO_2_ photocatalysts calcined at 200 °C exhibited decolorization of 88.94% and 63.83% respectively. At calcination temperature of 300 °C, decolorization of 95.06% and 81.70% was observed for 20N–TiO_2_ and 10N–TiO_2_ photocatalysts respectively. While photocatalysts calcined at 400 °C exhibited the lowest performance among all mol % nitrogen loading. The best combination of calcination temperature and nitrogen loading for maximum RB5 decolorization was observed for 20N–TiO_2_-300. Changes in photocatalytic activity of the aforesaid nitrogen-doped photocatalysts at different calcination temperatures can be ascribed with the availability of the active sites, loss or replacement of nitrogen by oxygen, photon absorption, electron–hole recombination rate, specific surface area and crystallite size [2,21,30,36,37,38].

#### 2.2.2. Adsorption of RB5 in Dark Condition

**Figure 7:**
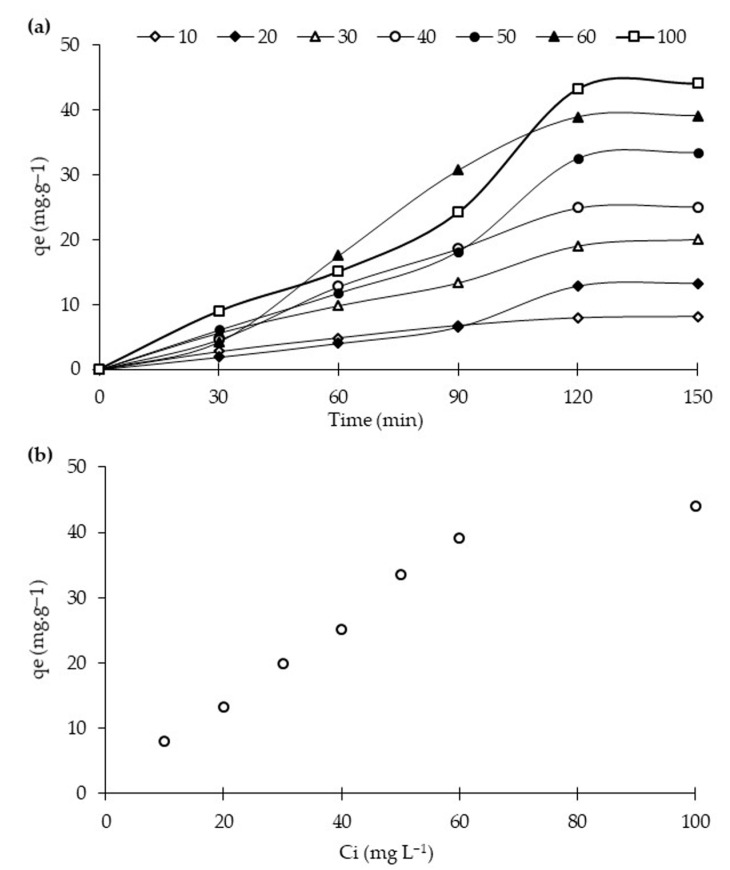
Adsorption capacities of 20N–TiO_2_-300 for RB5 at a variable initial increasing concentration as a function of contact time (**a**), and at equilibrium (**b**).

Dark reaction studies were carried out to check the adsorption capacity of the synthesized photocatalysts. Dye molecule distribution between the liquid and solid phase is determined through adsorption isotherms models, mainly Langmuir and Freundlich. Figure 7a shows the adsorption capacities of the best-performing photocatalyst, i.e., 20N–TiO_2_-300 reported above, for RB5 at an increasing concentration from 10 to 100 mg L^−1^ as a function of time. It is clearly shown from the figure that the RB5 adsorption capacities of 20N–TiO_2_-300 increased three times with the increasing solution concentration until all the adsorption sites are occupied and equilibrium achieved at 120 min. It is also exhibited in Figure 7b that maximum adsorption capacities at equilibrium (qe) for 20N–TiO_2_-300 photocatalyst as a function of RB5 initial concentration (Ci) were up to 60 mg L^−1^, and further increment in the RB5 initial concentration (Ci) exhibited a steady curve.

**Figure 8:**
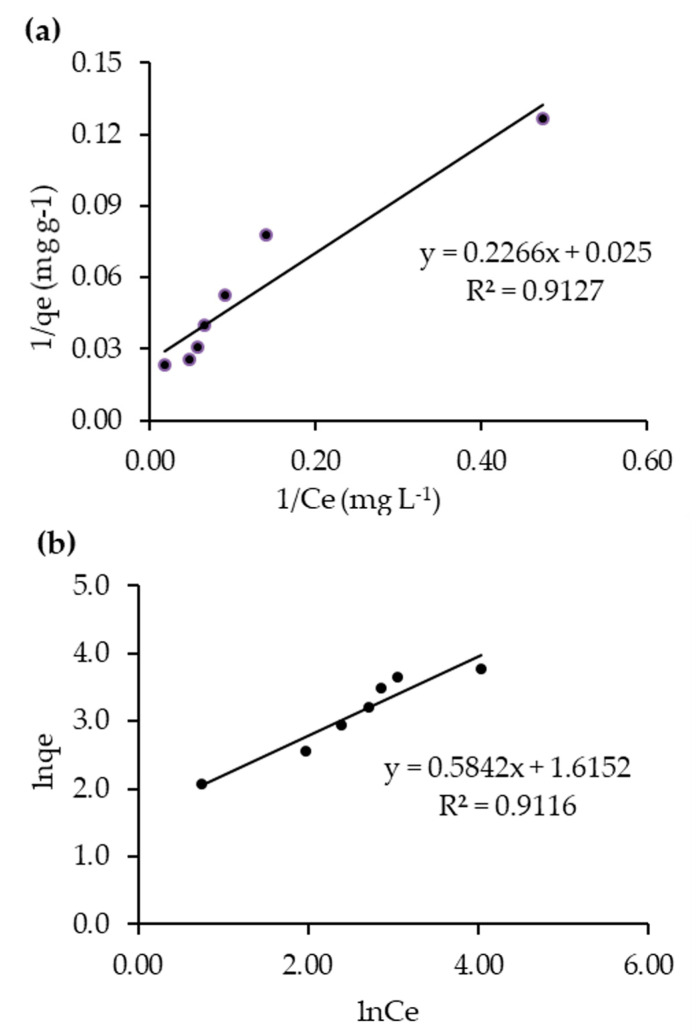
RB5 decolorization data fitness to (**a**) Langmuir and (**b**) Freundlich adsorption isotherms.

To investigate the adsorption mechanism of RB5 under dark conditions, the data fitted into the linear form of the Langmuir and Freundlich isotherm models. Figure 8a shows the fitness of Langmuir adsorption isotherm, where maximum adsorption capacity (q_m_, 40 mg g^−1^) and Langmuir adsorption constant (K_ads_, 0.1103 mg^−1^) for 20N–TiO_2_-300 were obtained from the plot 1/q_e_ vs. 1/C_e._

Freundlich plot of lnq_e_ vs. lnC_e_ is shown in Figure 8b. The relative adsorption capacity (K_F_) and heterogeneity factor (1/n) were found as 5.03 L g^−1^ and 0.58, respectively. According to previous reports, favorable adsorption occurs if the value of the heterogeneity factor (1/n) is found to be less than 1 [39,40]. It can be shown from Table 5 that the 1/n value obtained was displayed as 0.58 which supports the studied nanomaterial as favorable for RB5 adsorption. Based on the best fit of the isotherm models, RB5 dye adsorption on to the surface of 20N–TiO_2_-300, better described by the Langmuir adsorption isotherm, hence follows monolayer adsorption behavior, since the R^2^ for Langmuir and Freundlich (multilayer) isotherms were found to be 0.9127 and 0.9116 respectively. Previous studies have also suggested monolayer adsorption (Langmuir) of methyl orange dye onto the surface of the N–TiO_2_ photocatalyst [41,42] that acts as an electron donor, transferring electrons from its excited state into the conduction band of the photocatalyst under light irradiation [43], thus providing more active sites for adsorption of dye molecules. In addition, the identification of isothermal and hysteresis loop types can be a valuable starting point for characterizing a nanoporous adsorbent via BET analysis. Figure 8 shows that adsorption follows a type II isotherm, a standard form of isotherm which reflects uncontrolled multilayer–monolayer adsorption [44,45], indicating the completion of monolayer coverage and the start of the multilayer adsorption.

### 2.3. Effect of Different Reaction Parameters

#### 2.3.1. Effect of Photocatalyst Dose

**Figure 9:**
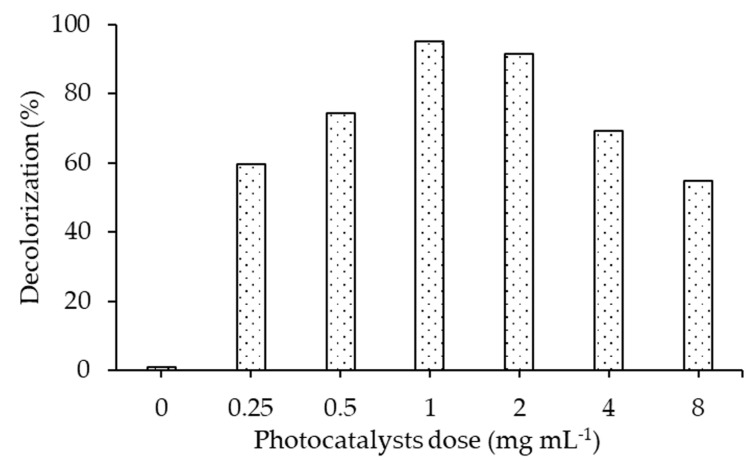
Effect of different photocatalyst dose on the decolorization of RB5 dye using 20N–TiO_2_ calcined at 300 °C, (Reaction conditions: dye conc. 30 mg L^−1^, reaction temperature 22 °C, pH 6.8).

Figure 9 demonstrates 60%, 95.06% and 54% decolorization of the RB5 dye using 20N–TiO_2_-300 photocatalyst doses of 0.25 mg mL^−1^, 1 mg mL^−1^ and 8 mg mL^−1^, respectively. These results show that increment in dosage ensures the availability of more active sites on the photocatalyst surface until it super passes a certain amount of photocatalyst. This also indicates that the screening effect can occur at a higher amount of photocatalyst, which covers the photoactive component of the catalysts and thus decreases the efficiency of the photodecolorization. Different factors like the light penetration, internal mass transfer and formation of agglomerates due to particle–particle interaction result in a reduction of the photodecolorization ability at higher photocatalyst doses [23,37,46,47]. Consequently, on the basis of our observations and recent reports [48,49], 1 g L^−1^ of photocatalyst dose was selected as the optimum dose for decolorizing RB5 azo dye to resolve the above-mentioned high-dose impacts.

#### 2.3.2. Effect of pH

**Figure 10:**
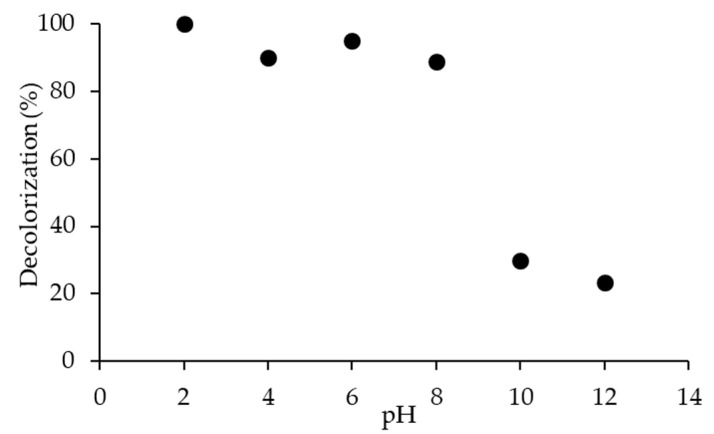
Effect of pH on the decolorization of RB5 dye using photocatalyst 20N–TiO_2_-300.

Figure 10 shows decolorization of 20N–TiO_2_-300 for RB5 dye with 100%, 90% and 96% at acidic pH 2, 4 and 6, while 88%, 29% and 23% at alkaline pH 8, 10 and 12 respectively. To determine the effect of pH, it is imperative to find the surface properties of the material. TiO_2_ is well-known for change in surface binding states with variation in pH. The point of zero charge (pH_pzc_) plays a prominent role in the effectiveness of the decolorization reaction by nanoparticles. The zero charges at the TiO_2_ surface were seen at pH_pzc_ 6.8, thus in acidic medium (<6.8), the surface of TiO_2_ is positively charged and alkaline medium (>6.8), is negatively charged [50]. Results corroborate this phenomenon, showing strong decolorization at pH < pH_pzc_, and the reverse was validated at pH > pH_pzc_. This also confirms the electrostatic interaction between the negatively charged anionic dye and positively charged TiO_2_ surface under acidic conditions, and similarly, the repulsion under alkaline conditions was expected [37].

#### 2.3.3. Effect of Initial Dye Concentration

**Figure 11:**
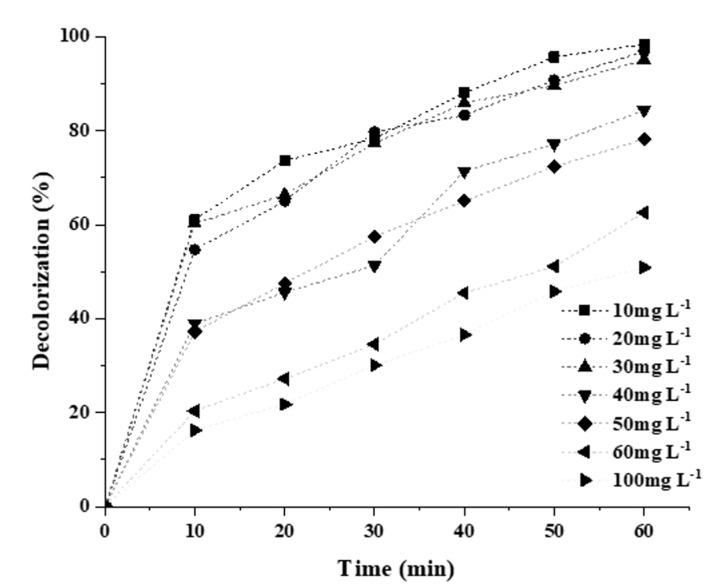
Effect of initial dye concentration on the % decolorization of 20N–TiO_2_-300 °C.

The effect of increasing the initial concentration of the dye from 10 to 100 mg L^−1^ is depicted in Figure 11. It can be observed from the figure that RB5 decolorization reduced from 100 to 50% when the concentration of contaminants increased from 30 to 100 mg L^−1^, respectively. This can be ascribed to the constant production of reactive radical species by the same amount of photocatalyst dose, and the intensity of light for a higher and lower concentration of RB5 dye, photon interception by RB5 molecules at high concentration and less number of photons reaches the photocatalyst surface, therefore, result in lower production of oxidization species and ultimately break free radical production as explained in Equation (1) [37,51].

(1) $${\mathrm{Dye}+\mathrm{OH}}^{\text{•}}↔{\mathrm{Dye}}^{\text{•}}{+H}_{2}O\to {\mathrm{OH}}^{\text{•}}+\mathrm{degradation}\mathrm{products}$$

### 2.4. Photostability of RB5 Azo Dye

**Figure 12:**
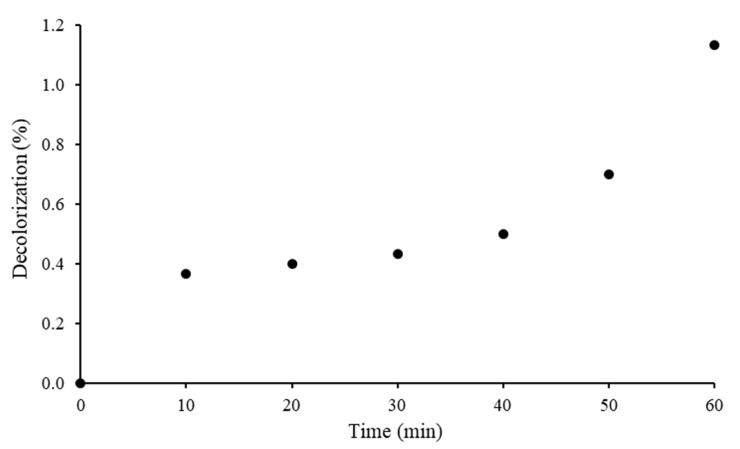
Photostability of the RB5 azo dye.

Photostability of RB5 dye was evaluated under the visible light without the addition of 20N–TiO_2_-300 photocatalyst. Figure 12 showed a negligible decolorization of RB5 by 1.1% dye after 60 min of irradiation under visible light. This insignificant decolorization of the RB5 dye can be attributed to the generation of superoxide. A similar phenomenon has been explained elsewhere [52] and no decolorization under visible light has been reported in the presence of a low concentration of oxygen. This shows that RB5 dye is stable to visible light and very little photolysis takes place in bulk solution.

### 2.5. Heterogeneous Photocatalytic Kinetic Studies

**Figure 13:**
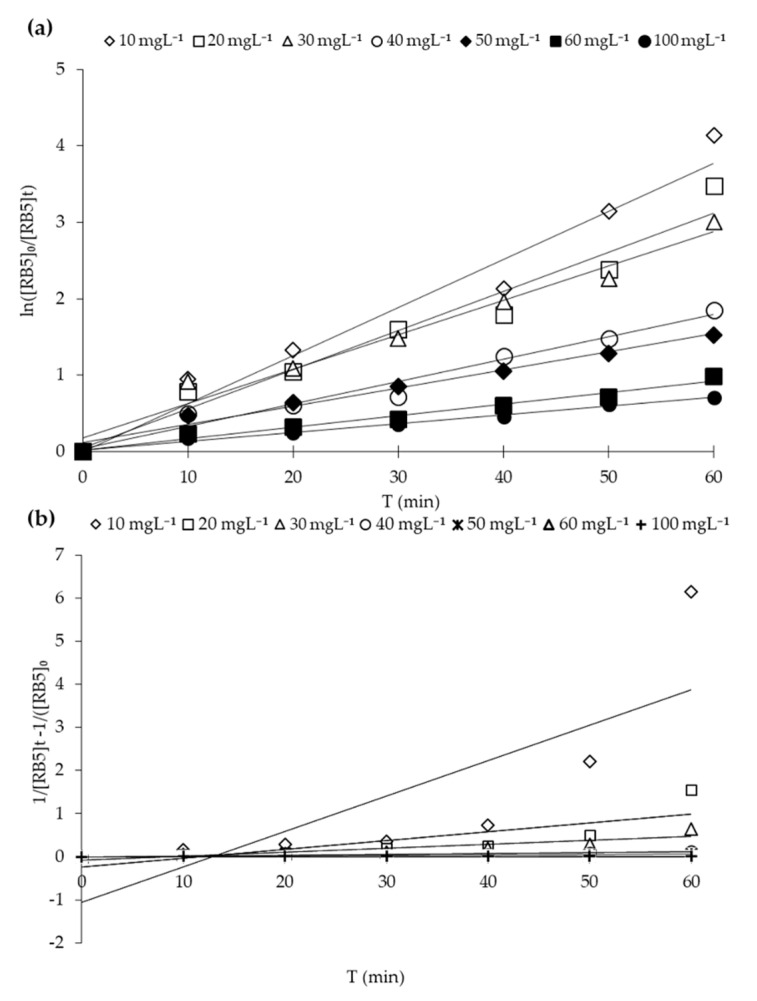
RB5 decolorization kinetics using 20N–TiO_2_-300 photocatalyst. (**a**) Pseudo first-order and (**b**) second-order.

Different kinetics models may be applied to assess the activity and decolorization (disappearance or mineralization) of pollutants [3,53]. Quantitative RB5 dynamical evaluation was carried out by fitting the decolorization data into kinetic models, e.g., pseudo-first-order (PFO) and second-order (SO) kinetic models. PFO kinetic plot (ln [RB5]e/[RB5]) versus the irradiation time (min) yielded a linear relationship, as shown in Figure 13a. The apparent first-order rate constant (K_app_) and R^2^ for the PFO model were calculated. PSO kinetic plot (1/[RB5]_t_-1//[RB5]_0_) versus the irradiation time (min) also yielded a linear relationship, as shown in Figure 13b. It is clearly illustrated from Figure 13 and Table 6 that the PFO model exhibited the best fit for RB5 adsorption with higher regression coefficients (R^2^) with increasing concentration of dye in the solution than SO model fitness. Therefore, the photocatalytic decolorization reaction of RB5 through 20N–TiO_2_-300 belongs to the pseudo-first-order reaction kinetics. Previous studies reported the much better fit of the decolorization of organic pollutants to PFO compared to other kinetic models [30,37,53,54,55].

**Figure 14:**
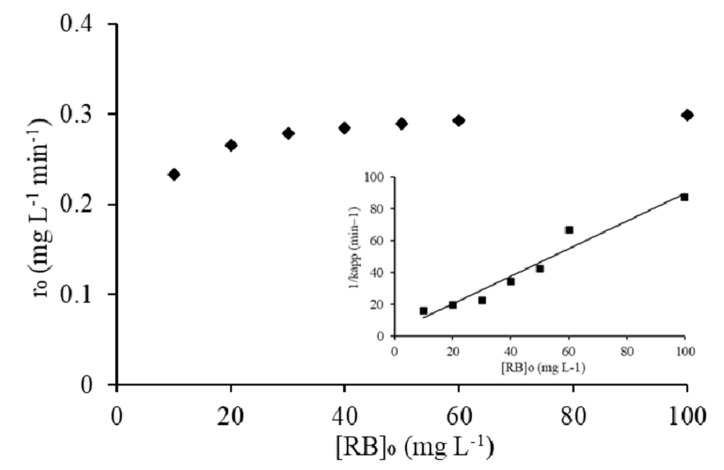
Effect of RB5 concentration on the initial rate of decolorization, inset: plot of reciprocal of apparent rate (K_app_) of decolorization against RB5 initial concentration.

The heterogeneous photocatalytic process is most commonly explained by the kinetic expression of the Langmuir-Hinshelwood isotherm model (L-H) shown in the inset of Figure 14. The coefficient, i.e., K_C_ (mg L^−1^ h^−1^) and L-H constant, i.e., K_LH_ (L mg^−1^), were determined through the Langmuir-Hinshelwood plot of 1/K_app_ versus [RB5]_o_. This model takes into account the assumption of both Langmuir and Freundlich and tells whether the photocatalytic decolorization is the dominant reaction mechanism with higher coefficient K_C_ and/or adsorption constant K_LH_. The model fitness yielded a straight line with R^2^ of 0.94, and the values of K_C_ and K_LH_ were calculated to be 1.15 mg∙L^−1^ h^−1^ and 0.30 L∙mg^−1^ from the slope (1/K_C_) and intercept (1/K_C_K_LH_) respectively.

From these results, the dominance of photocatalytic decolorization was persisted in comparison to RB5 adsorption onto 20N–TiO_2_-300 photocatalyst. If K_LH_ truly represents the adsorption of the dye on 20N–TiO_2_-300 surface, then Langmuir K_ads_ obtained should be the same as of K_LH_ in the L-H model; however, this could be observed under the ideal conditions. In this study, K_LH_ was found to be higher from the K_ads_. This difference can be ascribed to the availability of the active sites on the surface of photocatalyst and subsequent oxidation of dye molecules. As the active sites involved in RB5 adsorption under dark conditions may become available upon the decolorization of adsorbed dye molecules, resultantly increased the generation of radical species leading to high RB5 decolorization. Effective adsorption of RB5 was facilitated due to improved structural features of 20N–TiO_2_-300, for instance, increased pore volume (0.264 cm^3^ g^−1^) and pore diameters (21.3 nm). The maximum adsorption capacity (q_m_) predicted by the Langmuir isotherm model was 40 mg g^−1^. The value of R^2^ determined for the Langmuir isotherm model is higher than 0.9, which confirms that monolayer adsorption and chemical interaction of dye is predominant. Nitrogen adsorption–desorption BET type II with H3 hysteresis loop signifying 20N–TiO_2_-300 as a mesoporous substance and low hysteresis at higher relative pressure suggests capillary condensation. The effect of initial RB5 concentration [RB5]_o_ on the initial rate of decolorization, r_o_, is shown in Figure 14. It can obviously be seen that the rate of decolorization increases with an increasing initial concentration of RB5, which corresponds to the high value of K_LH_ of Langmuir–Hinshelwood adsorption model [56,57].

### 2.6. Photocatalytic Degradation Mechanism of RB5 Dye

**Figure 15:**
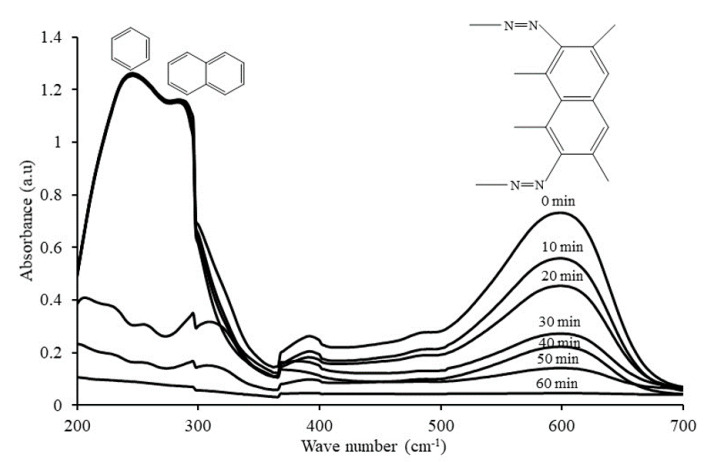
UV–visible absorption spectrums of RB5 azo dye at different time intervals.

**Figure 16:**
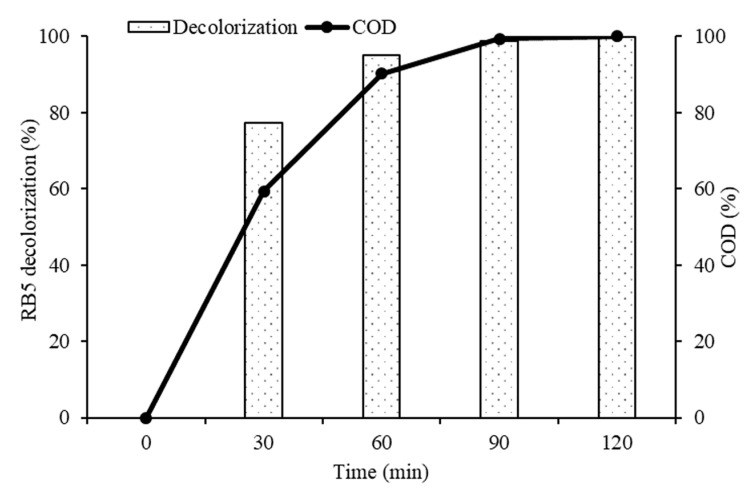
COD removal (%) at different time intervals using the 20N–TiO_2_-300 photocatalyst.

In order to understand and explain the photocatalytic degradation mechanism of RB5 azo dye using 20N–TiO_2_-300 under visible light irradiation, UV−visible spectrums of RB5 in the dye solution were recorded as a function of irradiation time as shown in Figure 15. Generally, RB5 dye exhibits three peaks at wavelengths of 244, 296 and 598 nm, which are attributed to benzene, naphthalene components and chromophore containing two azo groups linked by conjugated π-system, respectively [52]. The absorbance peak at 598 nm was used as the representative peak for the monitoring of RB5 decolorization. After 30 min of irradiation time, the disappearance of the representative peak was observed that could be due to the fragmentation of the azo links by oxidation, while all three peaks were disappeared by 20N–TiO_2_-300 photocatalyst within 60 min of light exposure. These results support the percent variation in COD over time. The COD analysis was performed to monitor the residual RB5 dye in solution after irradiation. Figure 16 showed a 90% reduction in COD with 60 min of irradiation. In addition, 20N–TiO_2_-300 photocatalyst was found to have completely mineralized RB5 dye within 120 min of visible light exposure. The photodegradation process can be best represented by two alternative routes, photolysis in the bulk solution as discussed earlier in Figure 9, and photocatalysis on the catalyst surface in Figure 16. Negligible decolorization was found in photolysis, i.e., 1.1%. This means that the degradation is carried out in the liquid bulk in the presence of photocatalyst.

### 2.7. Photocatalytic Disinfection Performance Evaluation

**Figure 17:**
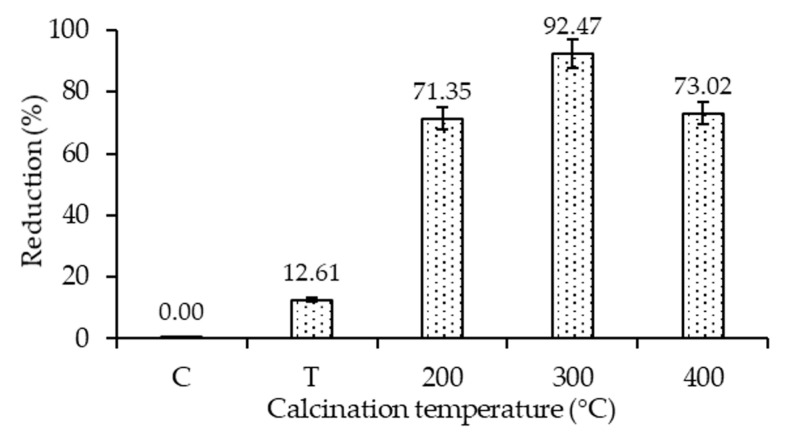
Antibacterial efficiency of the bare TiO_2_ (T) and 20N–TiO_2_ photocatalyst calcined at different calcination temperatures in 90 min of irradiation duration.

**Figure 18:**
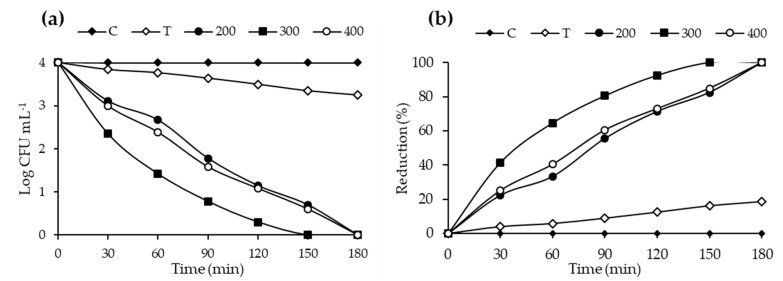
Kill time analysis of *E. coli* using control, bare TiO_2_ (T) and 20N–TiO_2_ photocatalyst calcined at different calcination temperatures (**a**) log CFU mL^−1^ and (**b**) Reduction (%).

The antibacterial studies were conducted using 20N–TiO_2_ photocatalyst against selected bacterial strain *E. coli* (ATCC-25922). Control (C), TiO_2_ (T) and 20N–TiO_2_ calcined at different calcination temperatures (200 °C, 300 °C and 400 °C) were used to study the antibacterial activity. 20N–TiO_2_-300 showed better photo-biocidal performance (92.47%) as shown in Figure 17 against tested bacterial strain. Kill time analysis was used to estimate the length of the time period required for maximum growth inhibition as shown in Figure 18a, significant decrease in the viability of the test pathogens over time was observed, while Figure 18b shows 100% growth reduction in 150 min of irradiation duration using 20N–TiO_2_-300 compared to other photocatalysts. This material showed a much better performance in a shorter time than previously reported for inactivation of *E. coli* in 420 min [11]. For another study, *E. coli* was inactivated in 90 min with N–TiO_2_, but the material was calcined at a high temperature of 600 °C [58]. Different factors for photo-biocidal activity were considered in these studies, including calcination temperature, a form of dopant (in the present case nitrogen) and improvement of physicochemical properties such as size, shape and specific surface area.

Studies have shown that material with smaller particle size helps to generate more ROS responsible for cytoplasmic constituent extrusion [59]. Furthermore, co-doping TiO_2_ with metal or non-metal exhibits excellent photo-biocidal activity [60]. In this study, the photo-biocidal activity for bare TiO_2_ was 12% while nitrogen doping improved photo-biocidal activity to 100% in 150 min under visible light irradiation. Larger specific surface area and lower particle size have also been reported as an important factor in bacterial inhibition because photocatalyst with a larger surface area tend to adhere more to the bacterial cell surface [60]. Unlike previous studies, 20N–TiO_2_-300 exhibited stronger biocidal activity in this study, even with the smaller surface area of 49.56 m^2^ g^−1^ and particle size between 11–44 nm, compared to bare TiO_2_ with a larger specific surface area of 66.31 m^2^ g^−1^. The reason can be the co-doping TiO_2_ with nitrogen and the presence of more crystalline anatase phase. As co-doping with nitrogen reduces the electron–hole recombination and can improve the photo-biocidal activity thus more ROS are possibly available for the inhibition of bacterial colonies.

Moreover, the antibacterial activity can be related to the dissolution mechanism or direct contact of the photocatalyst with bacterial cells [61]. Usually, the bacterial cell is negatively charged while the 20N–TiO_2_-300 is positively charged under the ambient conditions. The adhesion between the two surfaces is stronger due to the surface roughness, which in turns stimulate the photocatalyst attachment to bacterial cell thus the photocatalysts penetrates the cell structure and ultimately leads to cell death. These findings are supported by molecular docking studies, where strong evidence of entering TiO_2_ into active sites was found that ultimately lead to better photo-biocidal performance [62,63,64].

#### Molecular Docking Studies

**Figure 19:**
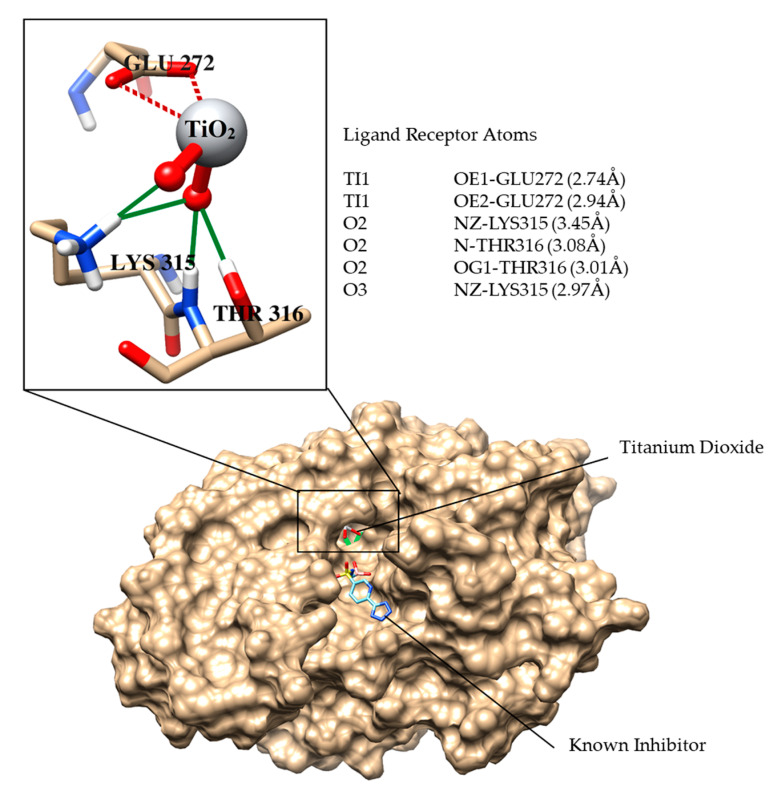
The binding mode of the known inhibitor (shown in cyan stick model) and TiO_2_ is shown in the active site of *E. coli* β-lactamase. The binding residues are presented in a tan stick model, Hydrogen bonds are demonstrated in green lines, metallic interactions between ligand and Glu272 are depicted in maroon dashed lines. The bond lengths between ligand and the interacting atoms of residues are written in parenthesis.

Nitrogen-doped TiO_2_ is well-known among nonmetals or anion dopants for its photocatalytic efficiency against many (organic and biological) pollutants. β-lactamases are produced by bacteria which provide multiple antibiotic resistances to bacteria by breaking the β-lactam ring of antibiotics, and thus deactivates antibiotic properties. Keeping in view the good bactericidal activity of 20N–TiO_2_-300 photocatalyst against *E. coli*, molecular docking studies were performed against the *E. coli* β-lactamase enzyme as a possible drug target. The docking score of the best-docked conformation of TiO_2_ was −7.6 kcal mol^−1^. Figure 19 shows the binding relationship between 20N–TiO_2_-300 Photocatalyst and active site residue. TiO_2_ entered deep inside the active site of β-lactamase and formed excellent interactions with the active site residues, including Lys315, Thr316 and Glu272. The dioxide groups mediated strong hydrogen bonding with the side chain of Lys315, the amino group of Thr316 and the side chain −OH of Thr316. Moreover, titanium was found to be engaged in metal–ligand interactions with the side chain of Glu272. The docking interactions and the bond length of interactions are summarized in Figure 19. The highly negative docking score and these significant interactions between the TiO_2_ and the β-lactamase confirm that the inhibition of β-lactamase could be the probable mechanism of bactericidal activity of 20N–TiO_2_-300 photocatalyst.

## 3. Material and Methods

### 3.1. Materials

The commercial dye reactive black 5 (RB5) azo dye, purchased from Sigma Aldrich, Steinheim, Germany, Titanium tetra-isopropoxide (TTIP) 98% purity was supplied by Daejung, China and used as TiO_2_ precursor, absolute ethanol 99% and glacial acetic acid 99% were purchased from Merck, Germany. Urea 98% was provided by Sigma, Germany, and used as the nitrogen precursor; deionized distilled water was produced in the laboratory using the B114 deionizer.

### 3.2. Photocatalysts Synthesis

**Figure 20:**
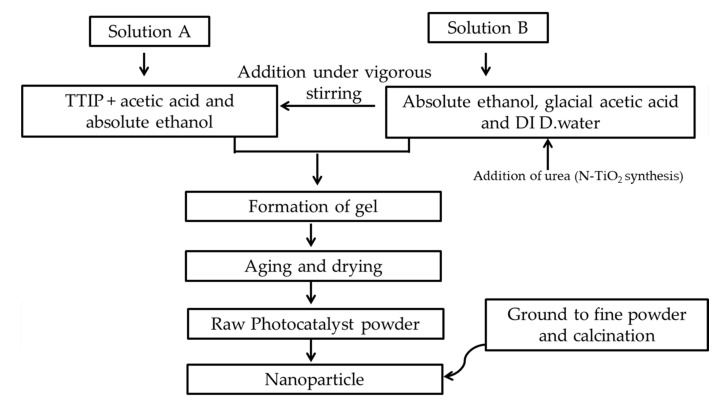
Flow diagram of photocatalyst synthesis.

TiO_2_ photocatalysts were synthesized through a modified sol-gel method [48]. Titanium precursor was added to absolute ethanol and termed as solution A. While another solution B was prepared by mixing deionized distilled water and acetic acid in absolute ethanol. 37 mL TTIP and 15 mL acetic acid were added into 60 mL absolute ethanol (solution A), while 0.662 g of urea, 10 mL acetic acid and 10 mL of distilled water were added in 14 mL absolute ethanol (solution B). Solution B was added dropwise to solution A under vigorous stirring. The solution was stirred until the formation of the gel. This was followed by 24 h of aging at ambient temperature and drying of the gel at 70 °C in an oven (UN 30, Memmert, Germany). To synthesize different mol% N–TiO_2_ photocatalysts (5, 10, 15, 20, 25 and 30), the desired amount of urea as nitrogen precursor (0.197, 0.417, 0.662, 0.938, 1.251, and 1.608 g) was added to solution B before being added to solution A, followed by the procedure explained above. Figure 20 shows the flow diagram of photocatalyst synthesis.

### 3.3. Photocatalyst Characterization

In order to determine the physicochemical properties, the best-performing photocatalysts were characterized for thermal stability using thermal gravimetric analyses (TGA-STA 8000, Waltham, MA, USA). Functional groups were detected through Fourier-transform infrared spectroscopy (FTIR-Alpha Bruker, Karlsruhe, Germany). Surface morphology was analyzed using scanning electron microscopy (SEM). Crystallite size and different phases of titania were identified using X-ray diffraction (XRD-Bruker, Billerica, MA, USA). Surface area and pore volumes were determined using Brunauer–Emmett–Teller analysis (BET- GEMINI VII 2390, Micromeritics Instrument Corp, Norcross, GA, USA). Bandgap was calculated using diffuse reflectance spectroscopy (DRS- UV-2600i, Kyoto, Japan). The samples were imaged in the SEM using a JEOL, JSM-6510LA, Tokyo, Japan, Analytical Scanning Electron Microscope model. XRD analysis was performed using an advanced diffractometer instrument equipped with a Cu K_α_ radiation source, at 40 kV and 40 mA. The pattern was scanned in the scanning angle (2θ) ranging from 10–80° at scan rate 2° min^−1^. The unknown components were identified by the standard diffraction data. Particle sizes (D) of the photocatalyst was estimated using the Scherrer formula in Equation (2) [26].

(2) $$D=\frac{K\lambda }{\beta \cos\theta }$$

The Scherrer constant (K) in the formula accounts for the shape of the particle and is generally taken to have the value 0.9 [65], λ (nm) represents the wavelength of rays, θ (radian) exhibit the diffraction angle of rays and β (radian) is the full width at half maximum (FWHM) of the reflection peak.

### 3.4. Photocatalytic Decolorization Studies

**Figure 21:**
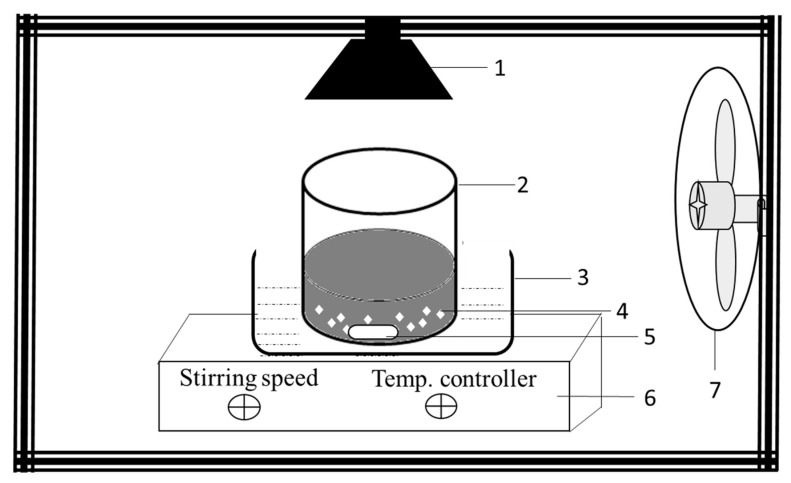
Scheme of the photocatalytic experimental setup; 1. Visible light source, 2. Reaction vessel 250 mL, 3. Water bath, 4. Photocatalysts, 5. Magnetic stirrer, 6. Stirring hotplate, 7. Cooling fan.

Photocatalytic decolorization studies were carried out for 30 mg L^−1^ RB5 with working initial pH 6.8 using a visible light source at room temperature using the photocatalytic experiment setup shown in Figure 21. Photocatalyst was weighed and mixed with distilled water and then ultrasonicated for 10 min using an ultrasonicator (FSF-020S Huanghua Faithful Instruments, Huanghua, China). For 30 mg L^−1^ final concentration, the desired amount of RB5 solution was added (with photocatalyst loading 1 mg mL^−1^) to make a total volume of 30 mL. The suspension was stirred using a stirring hot plate (DLAB MS7 H550-S, Riverside, CA, USA) at 200 rpm for 30 min in the dark and later illuminated for one hour with a visible light source at 25 cm. Halogen lamp (Hi Luminar, Bayern, Germany) has been used as a light source (500 W) with a light intensity of 30,798 lux, and additional details (Figure S1) provides light spectrum. Samples were collected at a specific time interval to monitor RB5 adsorption and removal during dark and light reactions. Experiments were also conducted without photocatalysts to check the photostability of the dye against the light source used.

### 3.5. Effect of Different Reaction Parameters

The effect of various reaction parameters on RB5 decolorization was measured, including contact time, pH, irradiation time, photocatalysts dose, and initial dye concentration. RB5 photocatalytic decolorization was monitored by measuring the solution absorbance from 400 to 800 nm using a PG instrument T80^+^ UV–visible spectrophotometer. A calibration curve was obtained beforehand using standard solutions with known RB5 concentrations (1, 10, 20, 30, 50, 60, and 100 mg L^−1^). The reaction samples were centrifuged before the absorbance measurement. The representative peak of RB5 (598 nm) was used for absorbance measurement. The color removal efficiency was calculated using the following expression.

(3) $$\mathrm{RB}5\;\mathrm{decolorization}\left(\%\right)=\left(\frac{C_{0}{-C}_{t}}{C_{t}}\right)\text{×}100$$

where C_0_ represents the initial dye concentration, and C_t_ indicates the residual dye concentration at the time, *t*.

### 3.6. Adsorption and Kinetic Study

The adsorption of the dye is an essential and prominent parameter in the photocatalytic process. In this study, the dye concentration decreased significantly when the adsorption was carried out in the dark. The best-performing photocatalyst, 20N–TiO_2_-300, were used to study the adsorption pathways. The RB5 experimental adsorption data of 20N–TiO_2_-300 were analyzed by well-established isotherms, namely Freundlich and Langmuir, and the goodness of fit was determined on the basis of coefficient of determination (R^2^). The linear transformation of Langmuir isotherm (Equation (4)) was used to calculate the value of q_m_ and K_ads_, the plot of 1/q_e_ and 1/C_e_ is constructed where K_ads_ can be derived from the slope (1/q_m_·K_ads_).

(4) $$\frac{1}{q_{e}}=\frac{1}{q_{m}}+\left(\frac{1}{K_{\mathrm{ads}}q_{m}}\right)\frac{1}{C_{e}}$$

The linear form of the Freundlich model is shown in Equation (5)

(5) $${\mathrm{lnq}}_{e}{=\mathrm{lnK}}_{F}+\frac{1}{n}{\mathrm{lnC}}_{e}$$

where q_e_ (mg·g^−1^) is the amount of dye adsorbed per unit weight of the photocatalyst at the equilibrium, q_m_ (mg·g^−1^) is the maximal adsorbed quantity, C_e_ (mg·L^−1^) indicate the residual concentration of the RB5 at equilibrium and K_ads_ (L·mg^−1^) is Langmuir adsorption constant. K_F_ [mg·g^−1^(L·mg^−1^)^1/n^] and n are the Freundlich constants related to the adsorption capacity and adsorption intensity, respectively.

### 3.7. Photocatalytic Kinetics

The Langmuir–Hinshelwood model [66] can be used to describe the relationship between the rates of the photocatalytic decolorization of RB5 dye in the presence of N–TiO_2_ photocatalysts as a function of irradiation time. The plot of 1/k_app_ against [RB5]_e_ gives a linear relationship between 1/k_app_ and [RB5]_e_. The values of K_c_ and K_LH_ can be determined from the slope (1/K_c_) and the intercept (1/K_c_K_LH_). To explain the effect of initial RB5 concentration on the initial rate of photodecolorization, the linear form of the Langmuir–Hinshelwood model can be written as:

(6) $$\frac{1}{r_{o}}=\frac{1}{K_{C}}+\frac{1}{K_{C}K_{\mathrm{LH}}}\times \frac{1}{{\left[\mathrm{RB}5\right]}_{e}}$$

Equation (6) translates the dependence of 1/r_0_ values on the respective 1/[RB5]_e_ values of RB5 concentration. Whereas, the values of K_c_ and K_LH_ are used to explain the effect of dye concentration on the equilibrium constant for the adsorption–desorption processes between the surface monolayer at N–TiO_2_ and the bulk solution.

### 3.8. Photocatalytic Disinfection Performance Evaluation

To check the photocatalytic disinfection ability, bactericidal activity was conducted using 20N–TiO_2_ photocatalyst calcined at three different calcination temperatures of 200 °C, 300 °C and 400 °C against *Escherichia coli* (ATCC-25922) as a model pathogen. The biocidal protocol explained elsewhere was followed for photo-biocidal performance [62]. The media and glassware were autoclaved before the experiment. Fresh broth cultures of *E. coli* were prepared, and 20N–TiO_2_-300 was added to mature bacterial culture in a glass vessel. The photocatalytic biocidal assay was conducted for 180 min with a known concentration in colonies forming unit (CFU) of *E. coli* (10^4^ CFU mL^−1^) and photocatalyst dose (1 mg mL^−1^) under visible light irradiation in photoreactor (Figure 21). The photocatalytic reactor was placed in the laminar flow to ensure the sterile state and prevent contamination. Known quantities of aliquots were extracted and plated at a regular interval of time, followed by incubation at 37 °C, and results were reported in the colony counter as CFU mL^−1^. For comparison, control experiments were also conducted in the dark and light. Results were also presented in terms of % reduction/inhibition before and after photocatalytic oxidation reaction (PCO) using the following formula:

(7) $$\mathrm{Percent}\;\mathrm{reduction}=\left(\frac{A-B}{A}\right)\text{×}100$$

where A indicates the number of viable bacteria before PCO and B denotes the number of viable bacteria after PCO.

#### Molecular Docking

The biochemical mechanism of the bactericidal activity of photocatalyst was rationalized by *Insilico* molecular docking approach. In order to perform docking, the three-dimensional X-ray crystal structure of *E. coli* β-lactamase (PDB ID: 4E3K, resolution = 1.43Å) in complex with known inhibitor (4-tetrazolyl pyridine sulfonamide boronic acid) was retrieved from RCSB Protein Data Bank [67] and docking was conducted on Molecular Operating Environment (MOE version 2014.09). β-Lactamases are enzymes found in outer membrane vesicles of β-lactam-resistant *Escherichia coli* that are able to inactivate the antibacterial properties of β-lactam antibiotics [68].

The protein file was prepared before docking, by adding hydrogen and partial charges via MOE’s Protonate 3D command. Water molecules and other heteroatoms, including phosphate molecules, were removed from the protein file. For docking, AMBER12: EHT forcefield was applied. The ligand file (TiO_2_) was prepared by MOE and Ti parameters (Mass = 47.8670, q = 0.9094, R = 1.5875, Eps = 0.1304, m = 12 and n = 6) were applied by AMBER12: EHT force field. Later, the default docking algorithm (triangle matcher) and scoring function (London dG) were used. After docking, each docked conformation was visualized, and the best orientation of ligand was chosen based on the binding interactions and docking score.

## 4. Conclusions

Different mass composition (mol %) of N–TiO_2_ were synthesized through a modified sol-gel method for the decolorization of double azo RB5 dye. The required physico-chemical properties of photocatalyst, i.e., 20N–TiO_2_-300 were successfully achieved. Effective adsorption of RB5 was facilitated due to improved physico-chemical features of 20N–TiO_2_-300, including shift of absorption spectrum, noticeable structural modification with reduced bandgap 2.95 eV, increased total pore volume (0.264 cm^3^ g^−1^) and average pore diameter (21.3 nm). XRD confirmed the dominant anatase phases, while no peaks of the dopant impurity were observed in the N–TiO_2_ nanomaterial due to lower nitrogen concentrations. SEM results exhibited the spherical but agglomerated morphologies. Nitrogen adsorption–desorption gave a BET type II with H3 hysteresis loop indicating 20N–TiO_2_-300 as a mesoporous material (average pore diameter = 21.3 nm) and small hysteresis at higher relative pressures indicates capillary condensation. The results obtained from screening photodecolorization studies indicated that 20N–TiO_2_-300 was very effective in photocatalytic decolorization of RB5 under visible light irradiation. The adsorption mechanism of RB5 in the dark reaction was studied by fitting the data into the linear form of the Langmuir and Freundlich isotherm models. The maximum adsorption capacity (q_m_, 40 mg g^−1^) and Langmuir adsorption constant (K_ads_, 0.1103 mg^−1^) was obtained from Langmuir isotherm for 20N–TiO_2_-300 while the Freundlich isotherm model depicts the relative adsorption capacity (K_F_) and heterogeneity factor (1/n) of 5.03 L g^−1^ and 0.58 respectively. Photocatalytic reaction kinetics of RB5 decolorization followed the pseudo-first-order kinetic model, which shows that the initial rate of RB5 decolorization was dependent of initial concentration [RB5]_o_. Moreover, in the current study, the constant K_LH_ (0.30 L∙mg^−1^) calculated for the Langmuir–Hinshelwood model was observed to be higher than that of Langmuir adsorption isotherm (K_ads_, 0.1103 mg^−1^) indicating the dominance of photodecolorization over adsorption in the reaction system. The photodegradation mechanism of RB5 dye was best-explained by the photostability to visible light, the disappearance of spectral peaks, and complete mineralization via COD monitoring. Moreover, good bactericidal activity 92.47% inhibition was obtained for best-performing photocatalysts 20N–TiO_2_-300 against *E. coli*. Molecular docking conformation was −7.6 kcal mol^−1^. The highly negative docking score and these significant interactions between the TiO_2_ and the β-lactamase confirm that the inhibition of β-lactamase by TiO_2_ could be the probable mechanism of bactericidal activity of 20N–TiO_2_-300 photocatalyst.

## Figures and Tables

**Table 1:** Thermogravimetric analysis N–TiO_2_ photocatalysts.

Photocatalyst	T Range (°C)	Weight Loss (%)
10N–TiO_2_	44–115	4.01
	116–799	17.85
15N–TiO_2_	44–115	3.83
	116–799	18.93
20N–TiO_2_	44–143	4.81
	144–799	25.72
25N–TiO_2_	45–154	12.44
	155–799	34.36
30N–TiO_2_	47–144	12.70
	145–799	37.47

**Table 2:** Crystallite size of synthesized photocatalysts.

Photocatalyst	FWHM β (Radian)	Crystallite Size D (nm)
15N–TiO_2_-300	0.024	6.28
20N–TiO_2_-300	0.026	5.73
TiO_2_ anatase	0.003	45.11

**Table 3:** Brunauer–Emmett–Teller (BET) surface area, pore volume, and pore diameter of different mol% N–TiO_2_ photocatalysts.

Sample	Surface Area (m^2^ g^−1^)	Total Pore Volume (cm^3^ g^−1^)	Average Pore Diameter (nm)
TiO_2_-300	66.31	0.113	6.85
5N–TiO_2_-300	71.09	0.126	7.12
10N–TiO_2_-300	69.32	0.126	7.30
20N–TiO_2_-300	49.54	0.264	21.3

**Table 4:** Effect of different calcination temperatures of the synthesized photocatalysts on RB5 decolorization.

Photocatalyst (mol %)	Decolorization (%)		
	200 °C	300 °C	400 °C
0N–TiO_2_	41.07	45.90	30.65
5N–TiO_2_	75.90	83.15	31.32
10N–TiO_2_	82.18	81.70	33.41
15N–TiO_2_	88.94	92.00	51.92
20N–TiO_2_	77.84	95.06	55.30
25N–TiO_2_	69.47	85.88	28.26
30N–TiO_2_	63.83	83.15	41.62

**Table 5:** Adsorption parameters and constants of isotherm modeling for 20N–TiO_2_-300 photocatalysts.

Isotherms	Plot	Parameters	R^2^
Langmuir	1/q_e_ vs. 1/C_e_	q_m_ = 40 mg g^−1^	0.9127
		K_ads_ = 0.1103	
Freundlich	lnq_e_ vs. lnC_e_	K_F_ = 41.23 mg g^−1^	0.9116
		1/n = 0.58	

**Table 6:** The apparent constant of PFO and SO kinetics of RB5 decolorization at different initial RB5 concentrations.

[RB5]_o_ (mg L^−1^)	Pseudo First Order		Second-Order	
	K_app_ (min^−1^)	R^2^	K_app_ (min^−1^)	R^2^
10	6.2 × 10^−2^	0.9556	8.22 × 10^−1^	0.6419
20	5.1 × 10^−2^	0.9577	2.04 × 10^−2^	0.6504
30	4.9 × 10^−2^	0.9716	9.00 × 10^−3^	0.7907
40	2.9 × 10^−2^	0.9705	2.10 × 10^−3^	0.8928
50	2.3 × 10^−2^	0.9810	1.10 × 10^−3^	0.9694
60	1.5 × 10^−2^	0.9801	4.00 × 10^−4^	0.9357
100	1.1 × 10^−2^	0.9915	2.00 × 10^−4^	0.9812

## Supplementary Materials

The following are available online, Figure S1: Light spectrum of halogen lamp (Hi Luminar-Germany) as a light source (500 W) with a light intensity of 30798 lux., Figure S2: Bandgap estimation of 20N-TiO_2_-300 from DRS spectra.
