# Supplementary material for: Photocatalytic Decolorization and Biocidal Applications of Nonmetal Doped TiO2: Isotherm, Kinetic Modeling and In Silico Molecular Docking Studies
Source: Molecules. 2020 Sep 29;25(19):4468. doi: 10.3390/molecules25194468 (PMC7583793; doi:10.3390/molecules25194468)
Supplement: Supplementary file 1 [file molecules-25-04468-s001.pdf]

Supplementary Information

Photocatalytic Decolorization and Biocidal Applications of Nonmetal Doped TiO_2: Isotherm, Kinetic Modeling and Insilco Molecular Docking Studies

Muhammad Saqib Khan [1], Jehanzeb Ali Shah [1], Muhammad Arshad [2], Sobia Ahsan Halim [3], Ajmal Khan [3], Ahson Jabbar Shaikh [4], Nadia Riaz [1], Asim Jahangir Khan [1], Muhammad Arfan [5], Muhammad Shahid [1], Arshid Pervez [1], Ahmad al Harasi [3*], Muhammad Bilal [1*]

[1] Department of Environmental Sciences, COMSATS University Islamabad, Abbottabad Campus, Abbottabad, 22060, Pakistan; muhammadsaqib@yahoo.com (M.S.K); jehanzeb360@yahoo.com (JAS); nadiariazz@gmail.com (N.R); asimjk@cuiatd.edu.pk (A.J.K); muhammadshahid@ciitvehari.edu.pk (M.S); pervez@cuiatd.edu.pk (A.P)

[2] Institute of Environmental Sciences and Engineering (IESE), SCEE, National University of Sciences and Technology, Islamabad, 44000, Pakistan; marshad@iese.nust.edu.pk

[3] Natural and Medical Sciences Research Center, University of Nizwa, P.O. Box 33, Birkat Al Mauz, Nizwa 616, Sultanate of Oman; sobia_halim@unizwa.edu.om (S.A.H); ajmalkhan@unizwa.edu.om (A.K); aharrasi@unizwa.edu.om (A.A)

[4] Department of Chemistry, COMSATS University Islamabad, Abbottabad Campus, Abbottabad, 22060, KPK, Pakistan; ahson@cuiatd.edu.pk

[5] Department of Chemistry, SNS, National University of Sciences and Technology, Islamabad, 44000, Pakistan; marfan@sns.nust.edu.pk

* Correspondence: mbilal@cuiatd.edu.pk Tel.: +92-992-383591 (M.B.), aharrasi@unizwa.edu.om (A.a.H); Tel: +968-25446328)

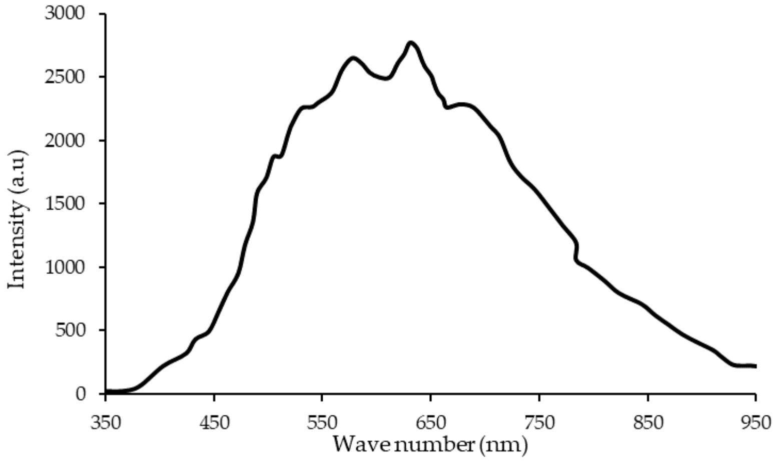

Figure S1. Light spectrum of halogen lamp (Hi Luminar-Germany) as a light source (500 W) with a light intensity of 30798 lux.

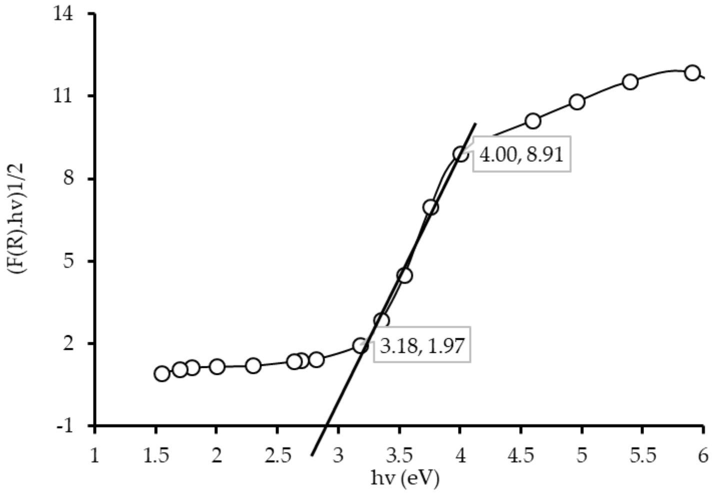

Figure S2. Bandgap estimation of 20N-TiO_2-300 from DRS spectra.

The bandgap of 20N-TiO_2-300 was obtained by extrapolating the tangent of the graph in the low energy range (hv) axis using,

$$y = mx + c \qquad (1)$$

x_1, x_2, y_1 and y_2 were obtained from the linear curve of 20N-TiO_2-300

x_1= 3.18 x_2= 4.00

y_1=1.97 y_2=8.91

To find out value of m,

$$m = \frac{y_1 - y_2}{x_1 - x_2} \qquad (2)$$

$$m = 8.4634$$

To find out value of c,

$$-c = y - mx$$

By putting the value of y_2, x_2 and m, the value of c is

$$c = -24.94$$

Therefore, inserting the value of m and c in equation 1

$$y = 8.4634x + (-24.94)$$

When y=0, $x = 2.95$

So, the estimated bandgap for 20N-TiO_2-300 is 2.95 eV
